# Digitale Transformation im stationären Altersbereich

**DOI:** 10.1007/s00391-020-01789-0

**Published:** 2020-10-01

**Authors:** Alexander Seifert, Friederike J. S. Thilo

**Affiliations:** 1 Zentrum für Gerontologie, Universität Zürich, Pestalozzistrasse 24, 8032 Zürich, Schweiz; 2 Hochschule für Soziale Arbeit, Fachhochschule Nordwestschweiz, Olten, Schweiz; 3 Angewandte Forschung und Entwicklung Pflege, Departement Gesundheit, Berner Fachhochschule, Bern, Schweiz

**Keywords:** Digitalisierung, Techniknutzung, Altersheime, Pflegeheime, Technologien, Digitalization, Use of technology, Retirement homes, Nursing homes, Technologies

## Abstract

**Hintergrund:**

Stationäre Altersinstitutionen durchlaufen derzeit einen digitalen Transformationsprozess, der durch die Orientierung an einer zunehmenden Digitalisierung von Arbeitsprozessen und der institutionellen Infrastruktur gekennzeichnet ist. Doch wie sieht die Vielfalt der eingesetzten technischen Lösungen in den Altersinstitutionen überhaupt aus, und wie wird der digitale Transformationsprozess von den Leitungspersonen der Institutionen bewertet?

**Material und Methoden:**

Die Befragung erfolgte als standardisierte Onlinebefragung bei stationären Alterseinrichtungen in der gesamten Schweiz. Befragt wurden die jeweiligen Leitungspersonen. Die Stichprobe bildeten 466 Institutionen.

**Ergebnisse:**

Etablierte Technologien, wie z. B. Fernseher, Kontakt- und Sturzmatten sowie Softwarelösungen im Verwaltungsbereich, werden fast flächendeckend eingesetzt, während Roboter zur Aktivierung der Klientel, Spielkonsolen oder telemedizinische Technologien nur sehr selten verwendet werden. Die befragten Personen sehen in der Anwendung technischer Hilfsmittel eher Vor- als Nachteile. Die größten Hindernisse, die bei der Einführung neuer Technik wahrgenommen werden, sind die damit verbundenen Kosten, fehlende Mitarbeiterkompetenzen und eine nichtvorhandene Infrastruktur. Die multivariate Analyse zeigt, dass der Digitalisierungsgrad in einer Einrichtung nicht nur von deren Größe abhängt, sondern auch von der Technikaffinität der leitenden Mitarbeitenden.

**Diskussion:**

Der Technologisierungsgrad in Altersinstitutionen ist unterschiedlich hoch, insgesamt aber eher ernüchternd niedrig, hinsichtlich der Verwendung neuer Technologien wie der Robotik oder Telemedizin. Barrieren für die digitale Transformation wurden aus der Befragung herausgearbeitet und diskutiert.

## Abstract (en)

**Background:**

Residential old age institutions are currently undergoing a digital transformation process, which is characterized by an orientation towards increasing digitalization of work processes as well as the institutional infrastructure. But what does the variety of technical solutions used in these old-age institutions look like and how is the digital transformation process assessed by the managers of these institutions?

**Material and methods:**

The survey was carried out as a standardized online survey of residential old age homes throughout Switzerland. The respective managers were interviewed. The sample was made up of 466 institutions.

**Results:**

Established technologies, such as television, contact and fall mats as well as software solutions in the administrative area are used almost everywhere, while robots for activating the clientele, gaming consoles or telemedical technologies are rarely used. Among those interviewed there were more people who saw advantages rather than disadvantages in the use of technical tools. The greatest obstacles that were perceived when introducing a new technology were the associated costs, a lack of employee skills and a nonexistent infrastructure. Multivariate analysis showed that the degree of digitalization in a facility depends not only on its size but above all on the technological affinity and rating of the senior staff.

**Conclusion:**

The degree of technology in old age institutions varies but overall it is rather sobering regarding the use of completely new technologies, such as robotics or telemedicine. Barriers to the increasing digital transformation were worked out from the survey and are discussed.

Stationäre Altersinstitutionen durchlaufen derzeit – so wie auch alle anderen Lebensbereiche – einen digitalen Transformationsprozess, der durch die Orientierung an einer zunehmenden Technologisierung der Arbeitswelt gekennzeichnet ist. Doch wie sieht der Digitalisierungsgrad in den Alters- und Pflegeheimen in der Praxis aus? Diese Kernfrage war leitend für die hier näher vorzustellende Befragung von Alterseinrichtungen in der Schweiz.

## Hintergrund und Fragestellung

Die Bedeutung moderner digitaler Technologien hat in den letzten Jahren nochmals stark zugenommen. Unser heutiges Leben ist geprägt von digitalen Infrastrukturen bzw. digitalen Technologien, die in einem zunehmend schnelleren Ablauf entwickelt werden und in allen Lebensbereichen an Bedeutung gewinnen [1]. Diese digitale Transformation findet auch im Betreuungs- und Gesundheitswesen statt. Dabei sind nicht nur Fragen der Automatisierung und Optimierung bestehender Prozesse relevant, sondern v. a. auch Aspekte wie Innovation, Flexibilisierung und Individualisierung, welche die heutige digital geprägte Welt begleiten [2]. Das Wissen, sog. digitale Kompetenzen, über die technischen Neuerungen und deren effizienten Einsatz, deren gezielten Nutzen und den Zugang zu ihnen ist unabdingbar, um den tatsächlichen Handlungsbedarf in Bezug auf die aufkommenden Herausforderungen in der Praxis zu erkennen [3].

Die Digitalisierung von Produkten, Dienstleistungen und Prozessen fordert in allen Branchen – und somit auch in den Altersinstitutionen – eine mehr oder weniger umfassende Neuausrichtung der Art und Weise, wie Unternehmen in diesem Bereich in Zukunft agieren bzw. ihre Arbeit mit digitalen Technologien unterstützen wollen [4, 5]. Dies bedeutet, dass die digitale Transformation nicht nur die technische Infrastruktur beeinflusst, sondern auch die Wertschöpfungskette sowie die Organisationsstruktur der Institution verändert [2]. Dies bedeutet z. B., dass innerhalb der stationären Alterspflege neue Technologien den Arbeitsalltag begleiten werden, dies aber auch neue technische Kompetenzen bei den Mitarbeitenden und teilweise auch bei den Bewohnenden nach sich ziehen, wenn z. B. ein Aktivierungs- oder Pflegeroboter bedient werden möchte [4]. Die erforderlichen Veränderungsprozesse greifen dabei tief in bestehende Ablauforganisationen ein. Folglich bedarf es einer sachlichen und zielorientierten Diskussion, wie Betreuungs- und Pflegeinstitutionen die zunehmende Digitalisierung und Technologisierung gestalten möchten. Zwangsläufig wird dabei die Frage aufkommen, wie die einzelnen Institutionen die digitalen Technologien in ihren Arbeitsabläufen einsetzen und wie sie diesem Einsatz gegenüberstehen. Momentan gibt es auf diese Fragen noch keine verlässlichen Antworten für die Schweiz.

Mit Blick auf die Altersinstitutionen muss bei der Betrachtung des digitalen Wandels eine besondere Aufmerksamkeit auf eine zusätzliche Dimension – nämlich die sozialen Einrichtungen – gerichtet werden, da diese Institutionen nicht selten in einem Spannungsfeld zwischen der betrieblichen Organisation und dem sozialen Auftrag stehen, den sie zu erfüllen haben. Soziale Einrichtungen orientieren sich an den Bedürfnissen ihrer Klient(inn)en und müssen daher abwägen, inwieweit technische Neuerungen die Klient(inn)en in ihrer Lebenswelt unterstützen oder beeinträchtigen [6]. Zudem müssen neue Technologien nicht nur vom Personal und der Klientel akzeptiert werden, sondern es sind auch technische Kompetenzen für deren Bedienung erforderlich [7]. Diese waren bislang im sozialen und im pflegerischen Arbeitsbereich jedoch kaum Bestandteil des Ausbildungs- und somit Anforderungsprofils [8, 9]. Parallel dazu bedarf es bei der Klientel – bedingt durch deren altersbedingte Vulnerabilität – einer besonderen Annäherung [10, 11].

Unklar ist, wie es tatsächlich um die digitale Transformation in den Betreuungs- und Pflegeinstitutionen in der Schweiz steht. Welche technischen Lösungen werden eingesetzt, und wie positionieren sich die Leitungen der Alterseinrichtungen zum Thema Digitalisierung? Diese Überlegungen waren Ausgangspunkt der vorliegenden Studie. Es soll hierbei gefragt werden: a) welche Techniken von den Alterseinrichtungen genutzt werden, b) welche Einstellung die Leitungen dieser Alterseinrichtungen hinsichtlich der Vor- und Nachteile der Techniken aufweisen und c) welche Faktoren den Digitalisierungsgrad (die eigene Positionierung der Institution im Vergleich zu den anderen Institutionen hinsichtlich der aktuellen Techniknutzung) erklären. Hierbei interessierte auch, inwieweit sich Institutionen in städtischen und ländlichen Regionen unterscheiden.

## Studiendesign und Untersuchungsmethoden

Im Rahmen der 2019 durchgeführten Mitgliederbefragung von CURAVIVA Schweiz, dem Branchenverband der Institutionen für Menschen mit Unterstützungsbedarf, wurden Fragen zum Themenbereich „digitale Transformation“ gestellt [12]. Die Grundgesamtheit der Befragung bildeten die 1469 in der Adressdatenbank von CURAVIVA Schweiz verzeichneten Mitglieder aus dem Bereich „Menschen im Alter“ (Alterseinrichtungen wie Alters- oder Pflegeheime). Sie wurden postalisch zur Teilnahme an der Onlinebefragung eingeladen (organisiert durch CURAVIVA Schweiz). Im Anschreiben wurden die Ziele der Befragung, die Teilnahmemodalitäten sowie die Freiwilligkeit der Teilnahme erläutert. Insgesamt haben an der Befragung 466 Institutionen im Bereich Menschen im Alter teilgenommen (Rücklauf von 31,7 %).

Die Fragen sollten von der jeweiligen Leitungsperson der Institution ausgefüllt werden, da diese meist die Anschaffung von technischen Neuerungen verantwortet und die strategische Ausrichtung der digitalen Transformation in der Institution konzipiert. Die Stichprobe beinhaltet *n* = 466 Personen (stellvertretend für je eine Institution) im Alter zwischen 28 und 65 Jahren (M = 52,8, SD = 7,42). Von den Befragten sind 37,5 % Frauen und 62,5 % Männer. Die meisten Einrichtungen befinden sich in der deutschsprachigen Schweiz (*n* = 387), 66 in der französischsprachigen und 13 in der italienischsprachigen Landesregion der Schweiz. Die Hälfte der Institutionen stammt aus einem ländlich geprägten Raum (52 %) (Tab. 1).

**Table 1:** 

Bewertungsbereich	Alle Institutionen (*n* = 466) Prozente bzw. Mittelwert (SD)	Nur städtisch oder Agglomeration (gültige *n* = 154)	Nur ländlich (gültige *n* = 167)	χ^2^-Test bzw. t‑Test (*p*)
*Person und Institution*				
Geschlecht (Frauen/Männer)	37,5/62,5 %	37,7/62,3 %	36,8/63,2 %	0,025 (0,875)
Alter (in Jahren)	52,82 (7,42)	52,57	53,13	0,656 (0,512)
Bildung^a^	2,82 (0,95)	2,98	2,78	−2,316 (0,021)
Anzahl der Betten in der Einrichtung	70,48 (43,51)	75,84	65,54	−2,128 (0,034)
*Technikbewertung*				
Technikaffinität^b^	4,13 (0,79)	4,19	3,96	−2,544 (0,011)
Technik bringt mehr Vor- als Nachteile^c^	3,95 (0,88)	4,06	3,76	−3,110 (0,002)
Klientel profitiert davon^d^	3,41 (1,12)	3,44	3,22	−1,686 (0,093)
Mitarbeitende profitieren davon^e^	4,11 (0,87)	4,11	4,04	−0,800 (0,424)
*Wichtigkeit von Technik im Bereich*^f^				
Sicherheit der Klientel	3,68 (0,60)	3,75	3,65	−0,890 (0,376)
Betreuung und Pflege	3,65 (0,56)	3,67	3,59	−0,702 (0,482)
Berichtswesen und Diagnostik	3,54 (0,64)	3,48	3,53	0,489 (0,626)
Unterhaltung und Aktivierung	3,43 (0,67)	3,57	3,30	−2,767 (0,006)
*Wahrgenommene Hindernisse*^g^				
Zu hohe Kosten	3,24 (0,74)	3,17	3,30	1,120 (0,228)
Fehlende Kompetenzen der Mitarbeitenden	3,12 (0,76)	3,16	3,06	−1,095 (0,275)
Fehlende Infrastruktur	3,11 (0,77)	3,11	3,17	0,622 (0,534)
Fehlende fachliche/technische Unterstützung	2,87 (0,78)	2,90	2,82	−0,760 (0,448)
Fehlende gesetzliche Vorgaben	2,69 (0,74)	2,74	2,67	−0,702 (0,484)
*Bedenken der Klientel und Mitarbeitenden *(Prozente „ja“)^h^				
Ängste bei der Bedienung	57,9 %	71,4 %	67,1 %	0,715 (0,398)
Sicherheitsbedenken (Sicherheit in der Bedienung und Zuverlässigkeit)	56,7 %	64,3 %	65,9 %	0,088 (0,766)
Datenschutzbedenken	55,4 %	61,0 %	62,9 %	0,115 (0,735)
Aufwand zum Erlernen der Bedienung	52,6 %	61,7 %	64,7 %	0,307 (0,580)
Angst vor Überwachung	44,4 %	54,4 %	49,1 %	0,951 (0,330)
Erkennen der Nützlichkeit von Technik	39,5 %	42,9 %	50,9 %	2,079 (0,149)
Ethische Bedenken	29,6 %	30,5 %	29,9 %	0,013 (0,910)
Bedrohung des eigenen Arbeitsplatzes	14,2 %	18,8 %	12,0 %	2,911 (0,088)

^a^Bildung (1 „Primarstufe“ bis 4 „Universität/Hochschule“)

^b^Technikaffinität (Einschätzungen auf einer 5er-Skala (1 „trifft gar nicht zu“–5 „trifft voll und ganz zu“), Mittelwert der 3 Aussagen („Das Bedienen moderner technischer Geräte fällt mir leicht.“ „Technischen Neuerungen sehe ich mit Zuversicht entgegen.“ „Hinsichtlich technischer Neuerungen bin ich sehr neugierig.“))

^c^Technik bringt mehr Vor- als Nachteile (Einschätzung der Aussage „Die Vorteile von Technik sind für meine Institution grösser als deren Nachteile“ auf einer 5er-Skala (1 „trifft gar nicht zu“ bis 5 „trifft voll und ganz zu“))

^d^Klientel profitiert davon (Einschätzung der Aussage „Sind Sie überzeugt, dass Ihre Klientel von technischen Neuerungen profitiert?“ auf einer 5er-Skala (1 „gar nicht überzeugt“ bis 5 „voll und ganz überzeugt“))

^e^Mitarbeitende profitieren davon (Einschätzung der Aussage „Sind Sie überzeugt, dass Ihre Mitarbeitenden von technischen Neuerungen profitieren?“ auf einer 5er-Skala (1 „gar nicht überzeugt“ bis 5 „voll und ganz überzeugt“))

^f^Wichtigkeit von Technik in unterschiedlichen Bereichen (Einschätzung der Aussage „Wie wichtig ist es Ihnen, dass in Ihrer Institution in den unten aufgeführten Bereichen moderne Technologie eingesetzt wird?“ auf einer 5er-Skala (1 „gar nicht wichtig“ bis 5 „sehr wichtig“))

^g^Wahrgenommene Hindernisse der Institutionsleitung (Einschätzung der Aussage „Inwiefern hindern die folgenden Punkte die Einführung von technischen Neuerungen?“ auf einer 5er-Skala (1 „hindert mich gar nicht“ bis 5 „hindert mich stark“))

^h^Von der Institutionsleitung vermutete Bedenken der Klientel und Mitarbeitenden (Einschätzung der Aussage „Wo sehen Sie aktuellen und künftigen Klärungsbedarf? Welche größeren Bedenken erkennen Sie bei der Klientel und den Mitarbeitenden?“ anzugeben mit „ja“ oder „nein“)

## Ergebnisse

**Figure 1:**
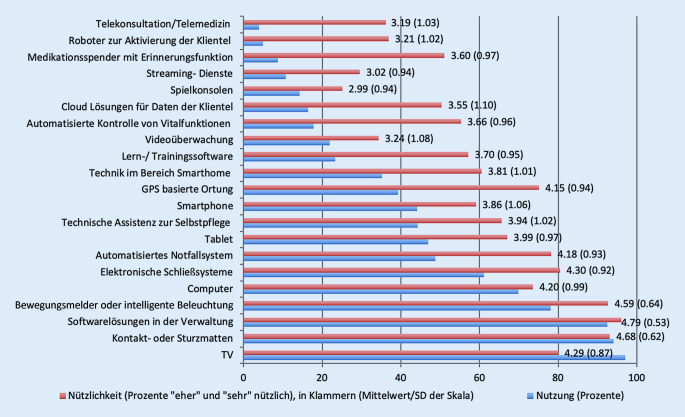

Wie in Abb. 1 zu sehen ist, wurden verschiedene Technologien und technische Geräte abgefragt, um herauszufinden, welche aktuell am häufigsten in den Alterseinrichtungen genutzt werden. Die Nutzung wurde dichotom erfragt (vorhanden/nicht vorhanden). Es ist zu erkennen, dass bestimmte Technologien, wie z. B. der Fernseher, Kontakt- und Sturzmatten sowie Softwarelösungen im Verwaltungsbereich, fast flächendeckend eingesetzt werden, dies jedoch nicht auf alle Technologien zutrifft. So werden beispielsweise Roboter zur Aktivierung der Klientel nur sehr selten verwendet, und auch die Telemedizin wird kaum zur Betreuung herangezogen. Auf der anderen Seite wird die (theoretische) Nützlichkeit („Nützlichkeit für die Arbeit vor Ort“; gemessen auf einer Skala von 1 „gar nicht nützlich“ bis 5 „sehr nützlich“) fast aller vorgestellten Technologien als hoch bewertet (Abb. 1); so werden auch Aktivierungsroboter und die Telemedizin, die vorher selten genutzt worden sind, nun mit einer mittleren Nützlichkeit bewertet.

Die Befragung erhob auch Informationen dazu, ob die Einrichtungen ihrer Klientel einen privaten Internetzugang gewähren. Von den befragten Institutionen gaben 14,6 % an, ihrer Klientel keinen Internetanschluss zur Verfügung stellen zu können bzw. zu wollen. Von den Institutionen, die einen Anschluss zur Verfügung stellen, gaben 33,7 % an, dass die Internetnutzung kostenpflichtig für die Klientel sei.

### Technikbewertung und Bedenken

Mit einem Mittelwert von 4,13 auf einer 5er-Skala mit 1 für eine niedrige und 5 für eine sehr hohe Technikaffinität weisen die befragten Personen im Durchschnitt eine relativ hohe Technikaffinität (Tab. 1: *Technikaffinität*) auf. Auch sehen mehr Personen Vor- als Nachteile in der Anwendung technischer Hilfsmittel in den Institutionen (Tab. 1: *Technik bringt mehr Vor- als Nachteile*). Im Vergleich werden aber die Vorteile der Techniknutzung eher für die Mitarbeitenden gesehen als für die Klientel (Tab. 1: *Klientel profitiert davon* und *Mitarbeitende profitieren davon*). Die Wichtigkeit, Technik in unterschiedlichen Bereichen einzusetzen, wird von teils, teils bis hoch eingeschätzt, wobei sich die Einsatzbereiche in puncto Wichtigkeit kaum voneinander unterscheiden (Tab. 1). Das größte Hindernis, das bei der Einführung neuer Technik in den Institutionen gesehen wird, sind die hohen Kosten – gefolgt von den fehlenden Kompetenzen der Mitarbeitenden und der fehlenden Infrastruktur (Tab. 1). Die befragten Institutsleitungen sahen bei der Klientel und den Mitarbeitenden Bedenken gegenüber neuer Technik; sie erwarten Ängste in Bezug auf die Bedienung sowie den Sicherheits- und Datenschutz und sind der Meinung, dass der Aufwand zum Erlernen der neuen Technik als zu hoch eingeschätzt werden könnte. Eine Bedrohung des Arbeitsplatzes befürchten die Mitarbeitenden – nach Angaben der Institutionsleitungen – wohl eher nicht (Tab. 1). Diese Aussagen unterscheiden sich kaum im Hinblick auf die räumliche Zuordnung (ländlich oder städtisch) (Tab. 1). Einzig beim Punkt Technikaffinität bzw. der Aussage, dass Technik mehr Vor- als Nachteile bringt, und dem Item „Wichtigkeit von Technik im Bereich Unterhaltung und Aktivierung“ ergeben sich statistisch signifikante Unterschiede. Hier sind die Institutionen aus dem städtischen Gebiet technikaffiner und offener für die Vorteile, die diese neue Technik mit sich bringt. Institutionen aus dem ländlichen Gebiet hingegen bewerten die Technologisierung des Unterhaltungs- und Aktivierungsbereichs als wichtiger.

### Digitalisierungsgrad

Um eine detailliertere Aussage darüber treffen zu können, inwieweit sich die Institutionen der Stichprobe in ihrer technischen Vielfalt voneinander unterscheiden, wurde ein Digitalisierungsgrad gebildet; dieser berücksichtigt, ob eine Institution mehr oder weniger von den abgefragten Technologien einsetzt, als dies im Durchschnitt über alle befragten Institutionen der Fall ist. Der Index (M = 0, SD: 0,16; Min/Max = −0,34/0,39) wurde durch die Ermittlung des Mittelwerts für jede Institution (Mittelwert (Anzahl der genutzten Techniken durch maximale Anzahl möglicher Techniken im Technikanwendungsbereich) pro Institution je Technikanwendungsbereich gewichtet nach (minus) dem Gesamtmittelwert aller Institutionen im jeweiligen Technikanwendungsbereich; alle Werte der jeweiligen Technikanwendungsbereiche wurden addiert) berechnet. Mit diesem Digitalisierungsindex wird sichergestellt, dass Technikbereiche, die für den Institutionstypus weniger wichtig sind, weniger ins Gewicht fallen, aber gleichzeitig Institutionen, die überdurchschnittlich viele Technologien einsetzen (also eine gewisse Vorreiterposition innehaben), mehr ins Gewicht fallen. Der Index besteht aus negativen und positiven Zahlen (−/+) und gibt an, ob sich eine Institution oberhalb oder unterhalb der Mitte (Mittelwert aller) befindet, also, ob die Institution einen tieferen oder höheren Digitalisierungsgrad hat.

Um herauszufinden, welchen multivariaten Einfluss die Soziodemografie, die Technikaffinität, die Aussage, dass die neue Technik mehr Vor- als Nachteile bringt, die Anzahl der Betten und die Zuordnung der Institution (städtisch oder ländlich) auf diesen Digitalisierungsgrad haben, wurde eine multivariate lineare Regression gerechnet. Abhängige Variable ist der Digitalisierungsgrad. Als unabhängige Variablen wurden in einem ersten Modell die Prädiktoren Geschlecht, Alter und Bildung eingebunden. In einem zweiten Modell wurden die subjektiven Bewertungen zur Technikaffinität und die Aussage, dass Technik mehr Vor- als Nachteile bringt, eingeführt, und in einem dritten Modell wurden die 2 Angaben zur Institution berücksichtigt: Anzahl der Betten und städtische Zuordnung. Die Analyse zeigt, dass neben der Größe der Einrichtung (Anzahl der Betten) v. a. die Technikaffinität und Technikbewertung (mehr Vor- als Nachteile) als Personenmerkmale der Leitungsebene das Vorhandensein eines gewissen hohen Digitalisierungsgrads miterklären. Dies bedeutet aber auch, dass nicht nur strukturelle Eigenschaften die Anschaffung von Technik begründen, sondern auch die persönlichen Einstellungen jener Personen, die diese Techniken heranholen. So sind Leitungspersonen, die selbst eine hohe Technikaffinität aufweisen, auch eher bereit, Technik im Arbeitsablauf einzusetzen (Tab. 2).

**Table 2:** 

Prädiktoren	Modell 1	Modell 2	Modell 3
	*B/Beta*	*B/Beta*	*B/Beta (95* *%-Konfidenzintervalle)*
Mann (ref. Frau)	0,052/0,171***	0,031/0,103	0,020/0,066 [−0,011/0,051]
Alter	0,001/0,053	0,002/0,104	0,001/0,063 [−0,001/0,003]
Bildung	0,037/0,242***	0,027/0,181***	0,018/0,120 [0,002/0,034]
Technikaffinität	–	0,035/0,181***	0,030/0,167** [0,010/0,049]
Technik bringt mehr Vor- als Nachteile für meine Institution	–	0,030/0,185***	0,030/0,180** (0,012/0,047)
Anzahl der Betten	–	–	0,001/0,254*** (0,000/0,001)
Stadt und Agglomeration (ref. ländlich)	–	–	0,007/0,025 (−0,022/0,037)
*F/df/p*	13,741/3/<0,001	15,855/5/<0,001	12,763/7/<0,001
*Korrigiertes R*^*2*^	0,090	0,164	0,211
*N (gültige)*	387	678	307

Abhängige Variable: Digitalisierungsgrad. Siehe Tab. 1 für Skalen der unabhängigen Variablen. Lineare Regression (Methode: Eingabe)

Signifikanzniveaus: ***p < 0,001, **p < 0,01, *p < 0,05

## Diskussion

Mit der vorliegenden Studie konnten Informationen zum digitalen Transformationsprozess und zu dessen Auswirkungen für den Altersbereich bei 466 Institutionen in der Schweiz ausgewertet werden. Die Studie ermöglichte einen ersten Einblick in die digitale Transformation im Altersbereich.

Die Institutionen verfügen über unterschiedlichste Technologien und setzen diese in unterschiedlichen Arbeitsfeldern ein. Computer oder Softwarelösungen zur Verwaltung sind am weitesten verbreitet; eher zukunftsgerichtete Technologien wie z. B. Roboter oder die Telemedizin kommen nur sehr vereinzelt vor. Sehr selten werden Roboter zur Aktivierung der Klientel oder die Telemedizin verwendet, obwohl deren Einsatz sowohl medial als auch im Forschungsdiskurs in letzter Zeit häufig besprochen wurde [13–16].

Weiter zeigte sich, dass die (theoretische) Nützlichkeit der vorgestellten Technologien als überwiegend hoch eingeschätzt wird, wobei gleichzeitig besonders zukunftsgerichtete Technologien noch selten genutzt werden. Dies kann damit erklärt werden, dass die befragten Leitungspersonen über eine relativ hohe Technikaffinität verfügen, wenn diese auch zwischen den Individuen stark variiert. Denkbar ist zusätzlich, dass die genannten Hindernisse – also Kosten, fehlende Infrastruktur, Datenschutz- und Sicherheitsbedenken sowie fehlende Kompetenzen der Mitarbeitenden – hier eine Rolle spielen. Diese identifizierten Hindernisse sind jedoch Voraussetzung und prioritäre Themen, wenn Institutionen sich auf den Weg der digitalen Transformation begeben möchten [2, 4].

Die Vielfalt der eingesetzten Technologien spiegelt sich in der Vielfalt der befragten Institutionen wider. Die multivariate Analyse zeigt, dass der Digitalisierungsgrad durch Merkmale der befragten Personen und strukturelle Gegebenheiten zu erklären ist. Genauer gesagt bestimmt die Größe der Institution (gemessen an der Anzahl der verfügbaren Betten) den Digitalisierungsgrad, jedoch hat auch die Technikaffinität der Leitungspersonen einen entscheidenden Einfluss – also deren Einstellung gegenüber dieser Technik i. Allg. und deren Einschätzung, ob diese Technik mehr Vor- als Nachteile für den Arbeitsprozess bietet. Diese Erkenntnis ist hoch relevant, denn die digitale Transformation benötigt klar definierte Rollen und solide Kompetenzen, um erfolgreich geführt werden zu können [2]. Die Größe der Einrichtung könnte hierbei ein Vorteil sein, da bestimmte Technologien bei einer gewissen Anzahl der Bewohnerschaft eher erst dann angeschafft werden, wenn die Kosten der Anschaffung auf die potenziellen Nutzerinnen bzw. Nutzer umgerechnet werden können.

Auch wenn die digitale Transformation mittlerweile den stationären Altersbereich erreicht hat, so sollte jede technische Innovation, die im Altersbereich eingesetzt wird, einer ethischen Diskussion unterzogen werden, um die Notwendigkeit ihrer Verwendung zu reflektieren und mögliche Nachteile frühzeitig zu besprechen – und dies im Idealfall nicht nur auf der Leitungsebene, sondern zusammen mit der Klientel und dem Mitarbeiterstamm [17, 18]. Hierzu sind weitere Studien über praxisnahe Evaluationen von Technologien, die im Altersbereich erfolgreich eingesetzt werden, notwendig, um Bewertungen und Empfehlungen aussprechen zu können. Die vorliegende Arbeit konnte hierbei erste Ergebnisse zur Istsituation liefern, um das Thema einzuordnen; jedoch bedarf es weiterer Forschung und vertiefter Befragungen mit einer jeweiligen praxisorientierten Einordnung für den Altersbereich.

### Limitationen

Der Branchenverband CURAVIVA erfasst den Großteil aller Schweizer Altersinstitutionen in ihrer Mitglieder-Adressbank, jedoch kann nicht ausgeschlossen werden, dass vereinzelte Einrichtungen nicht für die Umfrage eingeladen worden sind. Dies sollte bei zukünftigen Befragungen noch stärker berücksichtigt werden. Auch ist darauf hinzuweisen, dass an der Befragung die Leitungspersonen teilnehmen sollten; somit spiegeln die Antworten nicht unmittelbar die Perspektiven der Mitarbeitenden oder der Bewohnenden wider. Bei der Studie handelt es sich um eine Querschnittsuntersuchung; Veränderungen innerhalb einer Institution können daher nicht abgebildet werden. Für die weitere Forschung wäre es wünschenswert, einerseits individuelle Daten im Längsschnitt zu erheben, anhand derer die intraindividuelle digitale Transformation beobachtet werden könnte; andererseits wäre es für internationale Vergleiche erstrebenswert, wenn in weiteren Ländern spezifische Studien in Alterseinrichtungen durchgeführt würden. Darüber hinaus sollten auch Fragen der partizipativen Aushandlung von Technikeinführungen, Wünschen und Bedürfnissen der Klientel und der Mitarbeitenden berücksichtigt werden, für welche die vorliegende Studie keine personenbezogenen Daten erhoben hat.

## Fazit für die Praxis

- Die digitale Transformation ist auch im Altersbereich angekommen, eine flächendeckende Technikausstattung ist allerdings noch in weiter Ferne.
- Die Intensität der Technologisierung der Altersinstitutionen wird nicht nur von den strukturellen Gegebenheiten der Institutionen beeinflusst, sondern auch von der Einstellung der Leitungspersonen hinsichtlich der Vor- und Nachteile der neuen Technologien.
- Die Kosten für die Anschaffung dieser neuen Technologien, eine fehlende Infrastruktur, fehlende digitale Kompetenzen bei den Mitarbeitenden sowie sicherheitsbezogene und ethische Bedenken werden als Hindernisse für eine Einführung angegeben.

## References

[1] Snowdon A (2020). Digital health: a framework for healthcare transformation.

[2] Hess T (2019). Digitale Transformation strategisch steuern: Vom Zufallstreffer zum systematischen Vorgehen.

[3] Konttila J, Siira H, Kyngäs H (2019). Healthcare professionals’ competence in digitalisation: a systematic review. J Clin Nurs.

[4] Hülsken-Giesler M (2007). Pflege und Technik – Annäherung an ein spannungsreiches Verhältnis. Zum gegenwärtigen Stand der internationalen Diskussion. 2. Teil. Pflege.

[5] Heinze RG, Hilbert J, Naegele G, Olbermann E, Kuhlmann A (2016). Digitalisierung und Gesundheit: Transforming the Way We Live. Teilhabe im Alter gestalten.

[6] Kolland F, Wanka A, Gallistl V, Schroeter KR, Vogel C, Künemund H (2019). Technik und Alter – Digitalisierung und die Ko-Konstitution von Alter(n) und Technologien. Handbuch Soziologie des Alter(n)s.

[7] Kuhlmey A, Blüher S, Nordheim J, Zöllick J (2019). Technik in der Pflege – Einstellungen von professionell Pflegenden zu Chancen und Risiken neuer Technologien und technischer Assistenzsysteme.

[8] Hülsken-Giesler M, Daxberger S, Peters M, Wirth L-M (2019). Technikbereitschaft in der ambulanten Pflege. Pflege.

[9] Trübswetter A, Figueiredo L (2019). Digitalisierung in der deutschen Pflegeausbildung: Potenziale und Herausforderungen des AKOLEP-Projekts: Ein explorativer Zugang. Pflege.

[10] Remmers H (2019). Pflege und Technik. Stand der Diskussion und zentrale ethische Fragen. Ethik Med.

[11] Rüegger H (2016). Ethische Fragen zur Technikanwendung im Kontext der Betreuung und Pflege alter Menschen. Angew Gerontol Appl.

[12] Seifert A, Ackermann T (2019). Digitalisierung und Technikeinsatz in Institutionen für Menschen mit Unterstützungsbedarf. Eine Studie im Auftrag von CURAVIVA Schweiz.

[13] Becker H, Bendel O (2018). Robotik in der Gesundheitsversorgung: Hoffnungen, Befürchtungen und Akzeptanz aus Sicht der Nutzerinnen und Nutzer. Pflegeroboter.

[14] Baisch S, Kolling T, Rühl S (2018). Emotionale Roboter im Pflegekontext: Empirische Analyse des bisherigen Einsatzes und der Wirkungen von Paro und Pleo. Z Gerontol Geriatr.

[15] Hülsken-Giesler M, Daxberger S, Bendel O (2018). Robotik in der Pflege aus pflegewissenschaftlicher Perspektive. Pflegeroboter.

[16] Otto U, Brettenhofer M, Tarnutzer S (2015). Telemedizin in der älteren Bevölkerung. Ther Umsch.

[17] Kricheldorff C, König P, Klobucnik T (2017). Ethische Guidelines für Forschung und Entwicklung im Bereich Alter und Technik: Grundlagen für die Arbeit in Ethikkommissionen.

[18] Marziali E, Serafini JMD, McCleary L (2005). A Systematic Review of Practice Standards and Research Ethics in Technology-Based Home Health Care Intervention Programs for Older Adults. J Aging Health.

